# Effect of Hydroxychloroquine on QTc in Patients Diagnosed with COVID-19: A Systematic Review and Meta-Analysis

**DOI:** 10.3390/jcdd8050055

**Published:** 2021-05-13

**Authors:** Angelos Arfaras-Melainis, Andreas Tzoumas, Damianos G. Kokkinidis, Maria Salgado Guerrero, Dimitrios Varrias, Xiaobo Xu, Luis Cerna, Ricardo Avendano, Cameron Kemal, Leonidas Palaiodimos, Robert T. Faillace

**Affiliations:** 1 Jacobi Medical Center, Albert Einstein College of Medicine, Bronx, NY 10461, USA; salgad@nychhc.org (M.S.G.); varriasd@nychhc.org (D.V.); xux7@nychhc.org (X.X.); palaiodimosmd@gmail.com (L.P.); robert.faillace@nychhc.org (R.T.F.); 2 School of Medicine, Aristotle University of Thessaloniki, 54124 Thessaloniki, Greece; andreastzoumas@hotmail.com; 3 Section of Cardiovascular Medicine, Yale New Haven Hospital, Yale University School of Medicine, New Haven, CT 06510, USA; damiankokki@gmail.com (D.G.K.); avendano.ricardo@gmail.com (R.A.); 4 Montefiore Medical Center, Albert Einstein College of Medicine, Bronx, NY 10467, USA; lecu_2000@hotmail.com; 5 Leon H. Charney Division of Cardiology, New York University, New York, NY 10016, USA; qua125@gmail.com

**Keywords:** hydroxychloroquine, chloroquine, COVID-19, coronavirus, SARS-CoV-2, QTc, QTc prolongation, torsades de pointes

## Abstract

Background: Hydroxychloroquine or chloroquine with or without the concomitant use of azithromycin have been widely used to treat patients with SARS-CoV-2 infection, based on early in vitro studies, despite their potential to prolong the QTc interval of patients. Objective: This is a systematic review and metanalysis designed to assess the effect of hydroxychloroquine with or without the addition of azithromycin on the QTc of hospitalized patients with COVID-19. Materials and methods: PubMed, Scopus, Cochrane and MedRxiv databases were reviewed. A random effect model meta-analysis was used, and I-square was used to assess the heterogeneity. The prespecified endpoints were ΔQTc, QTc prolongation > 500 ms and ΔQTc > 60 ms. Results: A total of 18 studies and 7179 patients met the inclusion criteria and were included in this systematic review and meta-analysis. The use of hydroxychloroquine with or without the addition of azithromycin was associated with increased QTc when used as part of the management of patients with SARS-CoV-2 infection. The combination therapy with hydroxychloroquine plus azithromycin was also associated with statistically significant increases in QTc. Moreover, the use of hydroxychloroquine alone, azithromycin alone, or the combination of the two was associated with increased numbers of patients that developed QTc prolongation > 500 ms. Conclusion: This systematic review and metanalysis revealed that the use of hydroxychloroquine alone or in conjunction with azithromycin was linked to an increase in the QTc interval of hospitalized patients with SARS-CoV-2 infection that received these agents.

## 1. Introduction

Given the severity of the disease and paucity of safe and effective treatments for SARS-CoV-2 infection early in the course of the pandemic, physicians have resorted to drugs or compounds that have been used to treat other conditions in order to treat COVID-19 [1,2]. One of the first agents to be trialed and one that serves as the prime example of a repurposed agent with accelerated and widespread use is hydroxychloroquine [3]. It has been long used as an antimalarial and an immunosuppressive agent with a well-documented adverse event profile although cardiac toxicity is known to occur [1]. During the early stages of the pandemic, hydroxychloroquine and chloroquine, with or without the addition of azithromycin, were considered as potentially effective agents for patients with COVID-19 due to their antiviral and anti-inflammatory properties [4,5]. The interest in these agents arose from pre-clinical SARS-CoV-2 in vitro inhibition studies and small observational open-label randomized trials that demonstrated promise [2,6,7]. However, it is known that hydroxychloroquine, chloroquine and macrolide antibiotics may prolong repolarization as manifested as prolongation of the 12-lead ECG QT/QT corrected (QTc) interval [6,7]. Prolongation of the QTc interval can predispose patients to malignant ventricular arrhythmias including torsade de pointes [8,9]. In an effort to quantify the degree of QTc prolongation and the associated events of ventricular arrhythmias and torsade de pointes in patients with COVID-19 who were treated with hydroxychloroquine with and without azithromycin, multiple studies were published globally [10,11,12,13,14,15,16,17,18,19,20,21,22,23,24,25,26,27]. Our objective with this study was to systematically review the available literature, examining the association between the use of hydroxychloroquine with or without azithromycin in hospitalized patients with COVID-19 and QTc prolongation, as well as the incidence of ventricular arrhythmias and torsade de pointes. We also aimed to quantitatively synthetize the above evidence with a meta-analysis in order to reach conclusions regarding the safety of hydroxychloroquine with or without azithromycin in this patient population in terms of QTc prolongation and potential ventricular pro-arrhythmic effects. 

## 2. Materials and Methods

### 2.1. Literature Search and Inclusion Criteria

A systematic search was conducted in PubMed, Scopus, Cochrane and MedRxiv databases up to 31 July 2020. Two researchers (LP, AAM) independently searched the above-mentioned databases for potentially eligible studies. Whenever a disagreement occurred, a third investigator was involved (DGK) to reach a consensus. The following terms where used in different combinations to execute the literature search for our study: “hydroxychloroquine”, “chloroquine”, “plaquenil”, “QT”, “QT interval”, “QTc”, “QTc interval”, “arrhythmia”, “Torsades”, “Torsades de pointes”, “Torsade”, “Torsade de point”, “Covid”, “Covid-19”, “Covid19”, “Coronavirus”, “SARS-CoV-2”. Additionally, the investigators manually reviewed the reference lists of the included studies and previous reviews for additional potentially eligible articles. As prespecified inclusion criteria we used the following: (i) studies including patients hospitalized with COVID-19 that received at least one dose of hydroxychloroquine or chloroquine; (ii) studies that provided QT/QTc measurements in association with the use of hydroxychloroquine or chloroquine in the aforementioned population; (iii) studies that reported quantitative data for at least one of the outcomes of interest; (iv) studies in the English language. The prespecified exclusion criteria used included: (i) studies including potential overlapping populations with other included studies in this analysis, in an effort to avoid counting the same patients twice. In the case of studies with duplicated populations, the study with the larger sample was included in our review and analysis. 

The data supporting the findings of this study can be made available by the corresponding author upon reasonable request. This systematic review and meta-analysis was performed according to the preferred reporting items for systematic reviews and meta-analyses (PRISMA) guidelines [28]. 

### 2.2. Data Extraction and Outcomes

Two independent reviewers, mutually blinded (MS, AT), performed the data extraction from the selected studies to a prespecified form. If any disagreement occurred a third investigator (AAM) was involved to reach a consensus. Among the data extracted were the title of the study and the name of the first author, the month and year of publication, the city and country of the study, inclusion and exclusion criteria, patient numbers in the study and in each of the groups when applicable, baseline demographic characteristics, pertinent clinical characteristics and laboratory values, and outcomes of interest. The prespecified outcome was ΔQTc, QTc prolongation >500 ms that is commonly used as a cut-off in the literature as it is a well-known marker of high arrhythmic risk [29,30], and ΔQTc > 60 ms.

### 2.3. Risk of Bias Assessment 

**Figure 1:**
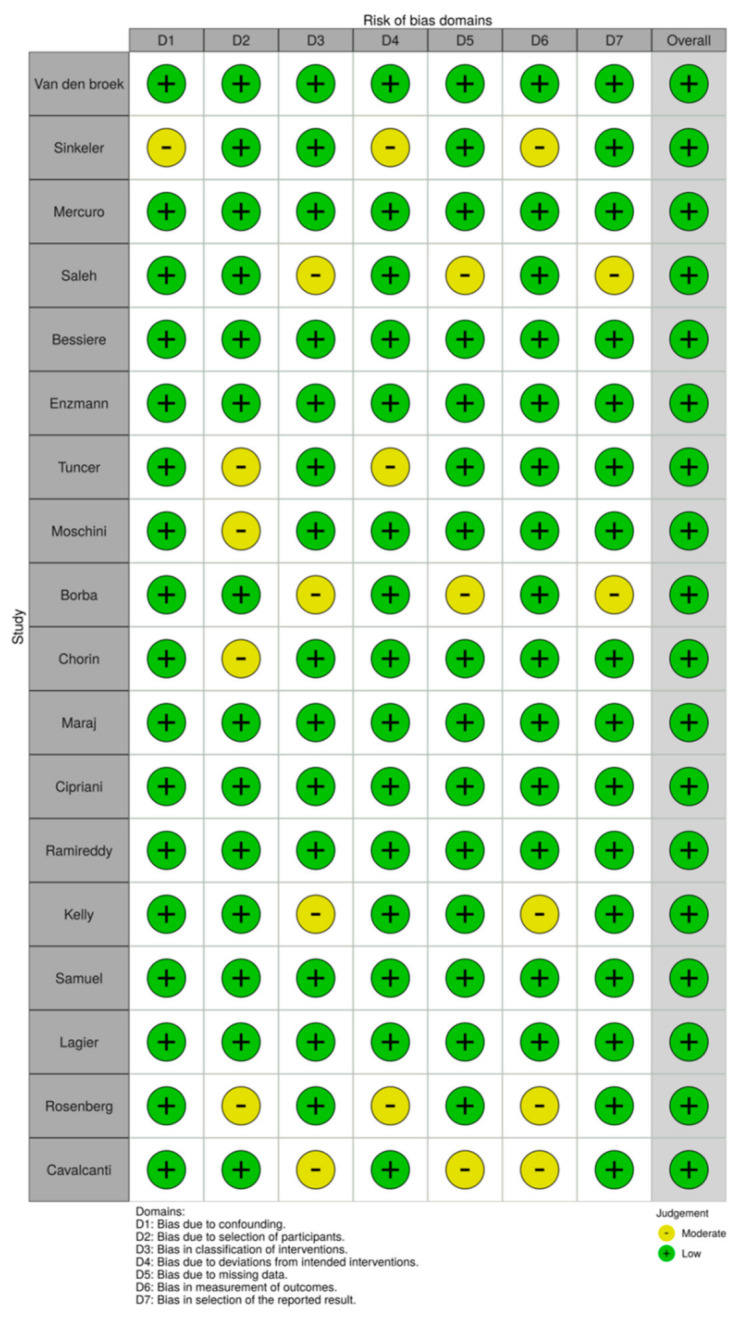
Risk of bias assessment.

The risk of bias was assessed by two blinded, independent reviewers (LP, AT) using the Robins-I tool for nonrandomized studies [31,32]. The following domains were evaluated: confounding, selection of participants, departure from intended interventions, missing data, measurement of outcomes, and selective reporting. Discrepancies in quality assessment were resolved via consensus. The ROBVIS (Risk-Of-Bias VISualization) tool was utilized to graphically display the results [33], (Figure 1).

### 2.4. Statistical Synthesis and Analysis

Descriptive statistics are presented as means and standard deviations (SDs) for continuous variables and number of cases (n) and percentages (%) for dichotomous and categorical variables. The cumulative incidence of patients with a QTc > 500 ms and a ΔQTc > 60 ms as well as the 95% confidence intervals were estimated and synthesized for each of the four treatment groups. Odds ratios (ORs) with the corresponding 95% CIs were used to compare the proportion of patients who developed a QTc and a ΔQTc value over the above threshold between the four treatment groups. Pooled estimates of the weighted mean difference (WMD) were calculated for outcomes describing continuous variables. The Higgins I-square (I^2^) statistic was used to assess heterogeneity among studies using a random effects model [34]. I^2^ > 75% indicated high heterogeneity [34]. Forest plots were used to graphically display the effect size in each study and the pooled estimates. A *p* < 0.05 was considered significant. The analyses were performed using STATA software (version 14.1; StataCorp College Station, TX, USA).

## 3. Results 

### 3.1. Search Results

**Figure 2:**
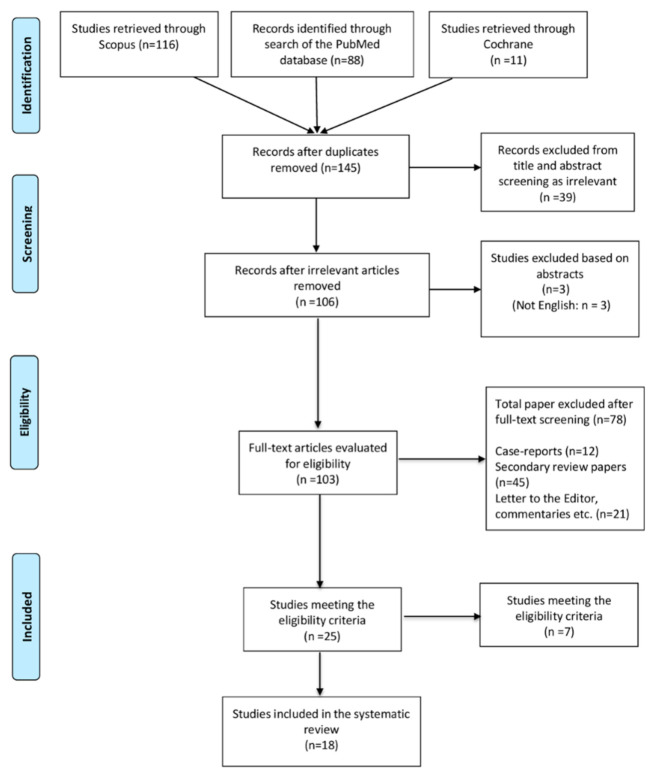
PRISMA flow diagram.

The literature search yielded 145 potentially relevant records after the duplicates had been removed. After screening the titles and abstracts, 103 studies were retrieved for full-text evaluation, and 18 studies [10,11,12,13,14,15,16,17,18,19,20,21,22,23,24,25,26,27] satisfied the predetermined search criteria and were included in the present meta-analysis as shown in the PRISMA (preferred reporting items for systematic reviews and meta-analyses) flow diagram (Figure 2).

### 3.2. Characteristics of Studies and Patients

Details about the included studies, including country of origin, number of patients and QTc correction formula used, are shown in Table 1. Detailed patient characteristics can be found in Table 2. The prevalence of baseline comorbidities such as hypertension, diabetes, coronary artery disease, obesity, heart failure, COPD/asthma, as well as the concomitant use of azithromycin and other QTc prolonging medications are also shown in Table 2.

### 3.3. Outcome Analysis

The mean QTc was increased in patients that received hydroxychloroquine alone (mean ΔQTc = 27.33 ms, SD: 50.05/mean max QTc = 472.57 ms, SD: 41.08), azithromycin alone (mean ΔQTc = 0.5 ms and SD: 40/mean max QTc = 464 ms, SD: 38, only one study), or the combination of the two (mean ΔQTc = 27.87 ms, SD: 36.230, mean max QTc = 467.50 ms, SD: 38.06). Moreover, the ΔQTc in patients that received hydroxychloroquine and azithromycin was found to be significantly increased compared to the ΔQTc of patients that received monotherapy with hydroxychloroquine alone (WMD = 16.06; 95% CI: 8.48, 23.65; I2 = 0.0%; *p* < 0.0001). 

**Figure 3:**
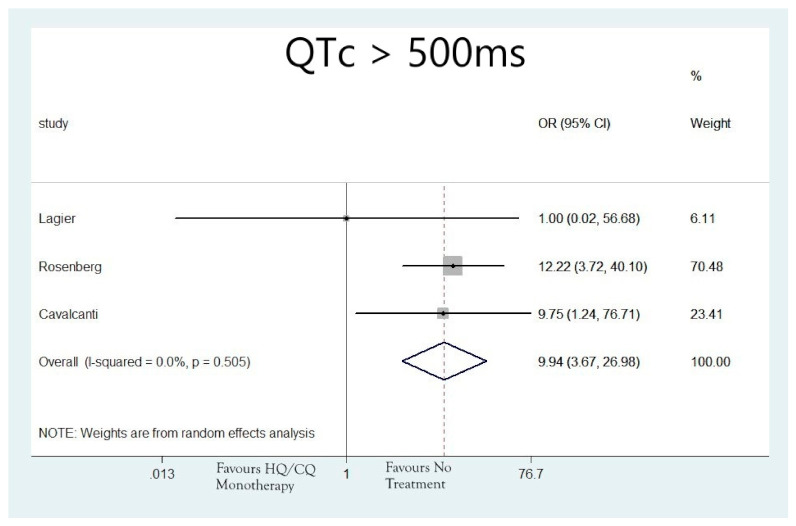
Risk of developing QTc > 500 ms in patients that received hydroxychloroquine monotherapy.

**Figure 4:**
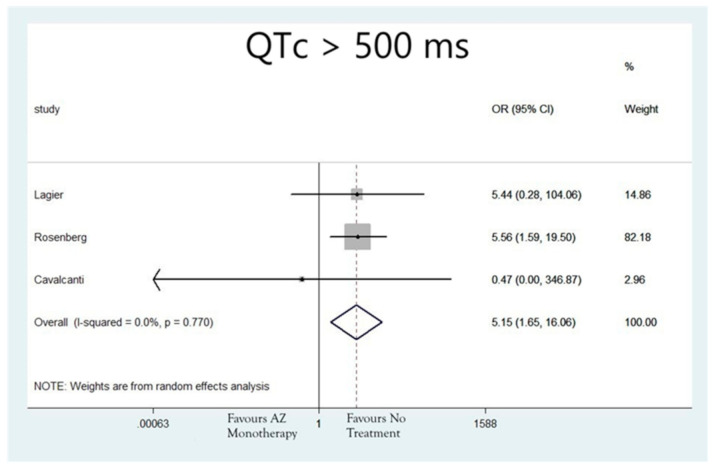
Risk of developing QTc > 500 ms in patients that received azithromycin monotherapy.

**Figure 5:**
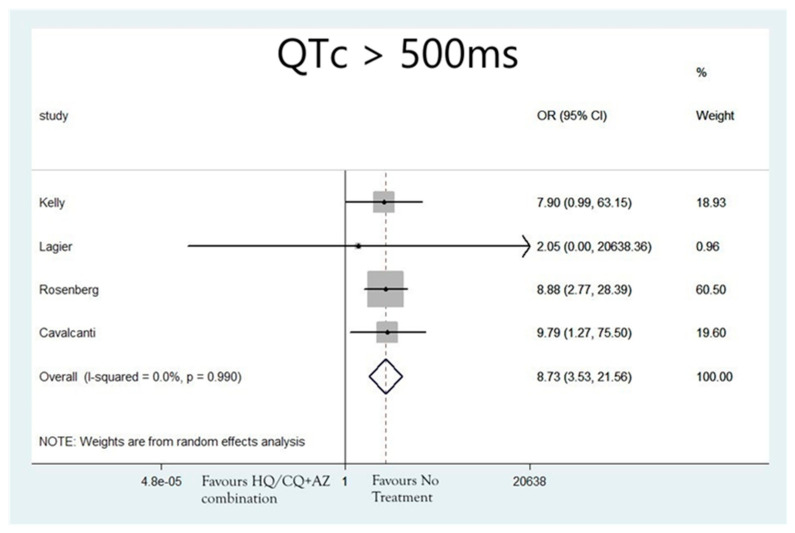
Risk of developing QTc > 500 ms in patients that received combination therapy with hydroxychloroquine and azithromycin.

The rates of patients that developed QTc > 500 ms were N = 152/1089 (13.96%), N = 217/5036 (4.31%), N = 22/381(5.77%) and N = 5/493 (1.01%) for patients treated with hydroxychloroquine as monotherapy, azithromycin as monotherapy, the combination of hydroxychloroquine and azithromycin or none of the aforementioned agents, respectively. The analysis also revealed that the groups of patients that received hydroxychloroquine alone (OR: 9.94; 95%CI: 3.67, 26.98; I2 = 0.0%; *p* = 0.00) (Figure 3), azithromycin alone (OR: 5.15; 95%CI: 1.65, 16.06; I2 = 0.0%; *p* = 0.005) (Figure 4), or the combination of the two (OR: 8.73; 95%CI: 3.53, 21.56; I2 = 0.0%; *p* = 0.0) (Figure 5), were associated with higher risk for developing QTc > 500 ms, compared to the group of patients that did not receive any of these agents. 

**Figure 6:**
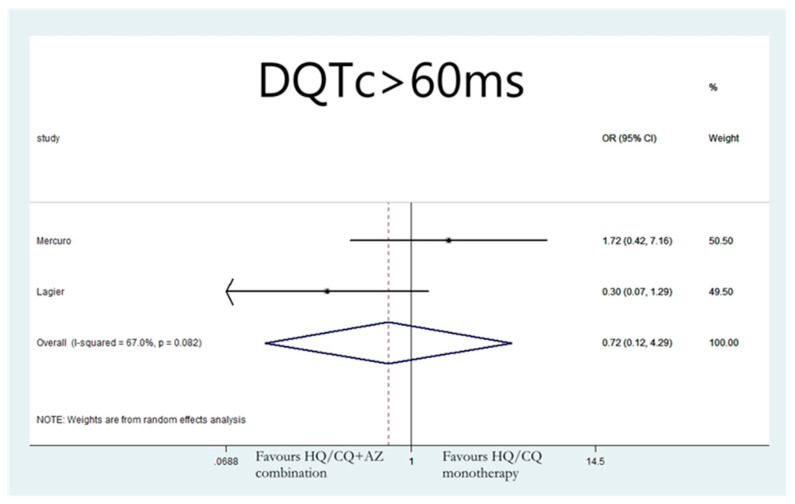
Risk of developing ΔQTc > 60 ms in patients that received combination therapy with hydroxychloroquine and azithromycin vs. hydroxychloroquine monotherapy.

**Figure 7:**
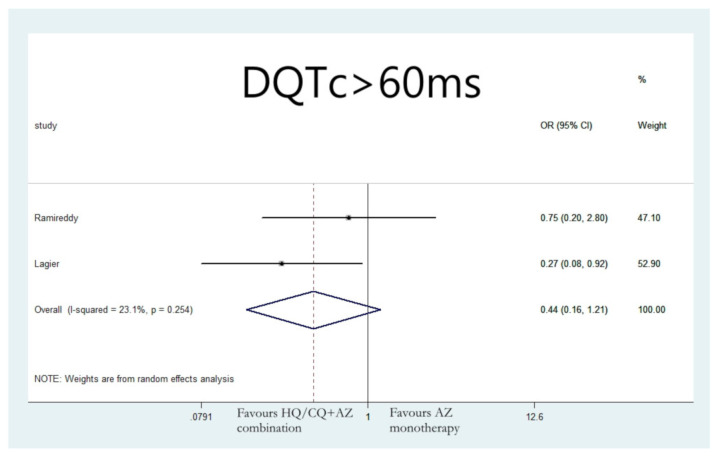
Risk of developing ΔQTc > 60 ms in patients that received combination therapy with hydroxychloroquine and azithromycin vs. azithromycin monotherapy.

Lastly, the group of patients that received combination therapy with hydroxychloroquine and azithromycin were not found to be significantly more likely to develop ΔQTc > 60 ms compared to patients receiving hydroxychloroquine monotherapy (OR: 0.72;95% CI: 0.12, 4.29; I 2 = 67%; *p* = 0.722) (Figure 6), or azithromycin monotherapy (OR: 0.44; 95% CI: 0.16, 1.21; I 2 = 23.1%; *p* = 0.112) (Figure 7).

### 3.4. Risk of Bias Assessment 

The outcomes of the Egger test were not consistent with publication bias. No evidence of publication bias was found for QTc > 500 ms when data from hydroxychloroquine monotherapy (*p* = 0.233), hydroxychloroquine and azithromycin combination (*p* = 0.199), or azithromycin monotherapy group (*p* = 0.376) were compared to the no treatment group.

## 4. Discussion

This study was a systematic review and metanalysis of the potential association between the use of hydroxychloroquine in hospitalized patients with COVID-19 patients and QTc prolongation. Our findings can be summarized as follows: (i) the QTc of patients that received hydroxychloroquine +/− azithromycin as part of their management for SARS-CoV-2 infection increased, (ii) combination therapy with hydroxychloroquine + azithromycin was associated with statistically significant increases in QTc, (iii) hydroxychloroquine alone, azithromycin alone, or the combination of the two was associated with increased number of patients that developed QTc prolongation > 500 ms.

Of note, hydroxychloroquine is currently no longer used for the treatment of patients with SARS-CoV-2 infection, however azithromycin is still recommended for the treatment of various infections. Our study also provides insight into the safety of using azithromycin as a monotherapy as well.

It is worth mentioning that the association between QTc prolongation and the development of clinically significant arrhythmias, including torsade de pointes, in this population is not well documented in the literature. In our systematic review, only a few events were reported in the included studies (Table 3). It is noteworthy, however, that based on real world data from the use of hydroxychloroquine and chloroquine as treatments for malaria or autoimmune diseases, these agents have been proven to be largely safe [35,36]. Interestingly, when used for patients with COVID-19, their effect on the QTc was reported to be exaggerated, rendering these patients more prone to arrhythmias [37,38]. QTc prolongation is a manifestation of a delayed ventricular repolarization that occurs as certain medications block the hERG potassium channel (product of the gene KCNH2), and as a result, block the delayed rectifier potassium current (IKr) [39]. The degree of QTc prolongation from hydroxychloroquine and its proarrhythmic sequelae in patients with COVID-19 might differ from previous data for a few reasons, some of which are highlighted below.

Given the long half-life of hydroxychloroquine, it would follow that patients receiving it for chronic conditions such as autoimmune diseases should be at higher risk, given the higher cumulative dose they usually receive [8,36]. However, hospitalized patients with COVID-19, are older and with significantly more baseline comorbidities. Moreover, they possess several risk factors which have been shown in the literature, to place them at higher-than-usual risk of ventricular arrhythmias, including electrolyte abnormalities -very important especially potassium and magnesium-, hypoxia, concomitant use of other QT-prolonging medications, underlying cardiovascular disease [40,41] and AKI (acute kidney injury). AKI in particular, could potentially contribute to the accumulation of the drugs in higher than usual concentrations in this patient population [42] and was independently associated with QTc prolongation in one of the early studies by Chorin et al. [13]. Patients with COVID-19 often concomitantly received azithromycin and/or other QTc prolonging agents. For instance, in the study by Ramireddy and colleagues, 74 of a total of 98 patients enrolled in the study were receiving at least two drugs known to prolong the QTc, in addition to hydroxychloroquine [21].

In addition, it remains unknown if COVID-19 itself has either a direct or an indirect effect on the myocardial repolarization and thus the QTc. Mechanistically, SARS-Cov 2 has been hypothesized to potentially affect the potassium rectifier channel by either directly impairing its function leading to an increase in the QTc interval or by sensitizing this channel to the impairing effects of known channel blockers such as azithromycin [43] and hydroxychloroquine [44]. Basic science data imply that both hydroxychloroquine and azithromycin have QTc prolonging potential via alternate mechanisms in addition to their possible effect on the potassium rectifier channel [45,46].

## 5. Strengths and Limitations

Notable strengths of this study include a thorough and updated review of the available literature, a strict methodology, a detailed and robust analysis and a relatively large number of patients with EKG recording data. In more detail, patients from three different continents, all with high prevalence of COVID-19 and hydroxychloroquine use were included in the analysis.

One of the major limitations of our study is the decreased rate of reported QTc monitoring in patients with COVID-19 not receiving hydroxychloroquine, thus potentially underestimating the effect of the virus itself and overestimating the risk of arrhythmogenesis associated with hydroxychloroquine. Moreover, regarding the outcome ΔQTc > 60 ms, not enough of the included studies reported this outcome in a way that would allow for a more meaningful and direct comparison among the treatment groups, thus the effects of hydroxychloroquine with or without the addition of azithromycin could be underestimated by our results. The number of doses of hydroxychloroquine or azithromycin varied significantly among the included studies. Additionally, the timing of the ECG in regards to the dosing of the medications was not clarified in most studies, as well as the follow up period was either short or not clearly stated in some of the studies. All the above could potentially have prevented us from detecting the full extent of QTc prolongation and its sequelae including, torsades de pointes, and/or deadly arrhythmias, and/or ventricular arrhythmias, something that could drastically change the risk estimates in rare adverse effects as the aforementioned. Finally, this is a systematic review and metanalysis of primarily observational studies and thus carries the inherent limitations of the respective studies, including potential unaccounted confounders.

## 6. Conclusions

This systematic review and metanalysis revealed that the use of hydroxychloroquine alone or in conjunction with azithromycin was linked to an increase in the QTc interval of hospitalized patients with SARS-CoV-2 infection that received these agents. The effects of this prolongation in respect to life-threatening arrhythmias and mortality was outside the scope of this study, thus further studies are needed to answer this clinically pressing question. While the limitations of our study are clear, we hope that the results of our study can add to the accumulating knowledge in the field of therapeutics for patients with SARS-CoV-2 infection.

## Figures and Tables

**Table 1:** Characteristics of included studies.

Study	Region/Country	Month, Year	Study Design	QTc Formula	Total N of pts	HQ/CQ Alone, N of pts	HQ/CQ + AZ, N of pts	AZ Alone, N of pts	No Treatment, N of pts
Van den Broek	Utrecht, Netherlands	April, 2020	R OBS	Bazett	95	95	0	0	0
Sinkeler	Tilburg and Amersfoort, Netherlands	July, 2020	R OBS (2 centers)	Postema and Wilde method	397	397	0	0	0
Mercuro	Massachusetts, USA	May 2020	R OBS	Bazett	90	37	53	0	0
Saleh	3 hospitals within Northwell Health System, NY, USA	April 2020	P OBS	Bazett	191	72	119	0	0
Bessiere	Lyon, France	May 2020	R OBS	Bazett	40	18	22	0	0
Enzmann	Fargo, ND, USA	June 2020	R OBS	NR	75	9	66	0	0
Tuncer	Istanbul, Turkey (pediatric only)	June 2020	R OBS	Bazett	21	2	19	0	0
Moschini	Cremona, Italy	July 2020	R OBS	Bazett	113	61	52	0	0
Borba	Manaus, Brazil	April 2020	P single-center	Fridericia	81	0	81	0	0
Chorin	NY, USA (NYU Langone Health); Milan, Italy	May 2020	R OBS	Bazett	251	0	251	0	0
Maraj	New Haven, USA	May 2020	R OBS	Bazett	91	0	91	0	0
Cipriani	Padua, Italy	May 2020	R OBS	Bazett and Fridericia (if HR > 100)	22	0	22	0	0
Ramireddy	California, USA	May 2020	R OBS	Bazett and Fridericia	98	10	61	27	0`
Kelly	Dublin, Ireland	July 2020	R OBS	NR	134	0	82	0	52
Samuel	NY, USA (Cohen Children’s Medical Center, pediatric only)	July 2020	R OBS	Bazett	36	16	9	0	11
Lagier	Marseille, France	June 2020	R OBS	Bazett	3737	101	3337	137	162
Rosenberg	NYC, Nassau County, Suffolk County, Westchester County, USA	May 2020	R multicenter OBS	NR	1438	271	735	211	221
Cavalcanti	Brazil	July 2020	P multicenter OBS *	NR	269	89	116	6	58

**Abbreviations:** R: retrospective, P: prospective, OBS: observational, N of pts: number of patients, QTc: corrected QT, NY: New York, NR: not reported. * Data were retrieved exclusively from an as treated analysis of the safety population of the non-blinded trial (NCT04322123), only for those patients with available electrocardiographic data, without randomized selection and adjusting for missing data.

**Table 2:** Baseline characteristics of patients per included study.

Study	N of Participants (Total)	Age	Male n (%)	BMI	Diabetes n (%)	HTN n (%)	CAD n (%)	CHF n (%)	Asthma/COPD n (%)	CKD II-IV or ESRD n (%)	Other QTc Prolonging Agents n (%)
Van den Broek	95	65	63 (66)	NR	NR	NR	11 (12)	9 (11)	NR	NR	NR
Sinkeler	397	67.8 (12.5)	262 (66)	28.5 (5.6)	NR	NR	32 (8)	32 (8)	NR	116 (29.2)	106 (27)
Mercuro	90	60.1 (16.7)	46 (51.1)	31.5 (6.6)	26 (28.9)	48 (53.3)	10 (11.1)	9 (10.0)	18 (20.0)	NR	90 (100)
Saleh	191	58.6 (9.1)	115 (60.2)	28.2 (2.8)	65 (32.3)	121 (60.2)	23 (11.4)	15 (7.9)	30 (15.7)	10 (5.2)	81 (42.4)
Bessiere	40	66.7 (11.9)	32 (80)	28.7 (5.9)	16 (40)	23 (57.5)	NR	NR	NR	NR	20 (50)
Enzmann	75	56	43 (57.3)	NR	22 (28.7)	NR	NR	8 (10.7)	22 (29.3)	4 (5.3)	NR
Tuncer	21	14.16	9 (42.8)	23.7	NR	NR	NR	NR	2 (9.5)		0 (0)
Moschini	113	67.7 (9.6)	85 (75)	NR	16 (14)	32 (28)	NR	NR	4 (3.5)	6 (5.3)	61 (53.9)
Borba	81	51.1 (13.9)	61 (75.3)	28.6 (6.4)	14(25.5)	25 * (45.5)	NR	NR	NR	NR	81 (100)
Chorin	251	64.3 (20)	188 (75)	NR	67 (27)	135 (54)	30 (12)	8 (3)	18 (7)	28 (11.1)	73 (29)
Maraj	91	62.7 (15.1)	51 (66)	NR	26 (29)	42 (46)	13 (14)	NR	6 (7)	25 (27.4)	38 (42)
Cipriani	22	63.3 (10.37)	18 (82)	28.3 (4.4)	6 (27)	12 (55)	NR	NR	1 (5)	1 (5)	0 (0)
Ramireddy	98	62.3 (17)	60 (61)	27.8 (6.6)	22 (22)	59 (60)	NR	20 (20.4)	25 (26)	14 (14.3)	74 (75.5)
Kelly	134	66.04	51 (38)	NR	NR	NR	NR	NR	NR	NR	3 (2.2)
Samuel	36	12.6 (6)	20 (55.5)	NR	NR	0 (0)	0 (0)	NR	3 (8)	NR	2 (5.5)
Lagier	3737	45.3 (16.8)	1704 (45.6)	NR	312 (8.3)	561 (15)	NR	NR	338 (11)	NR	45 (1.2)
Rosenberg	1438	NR	858 (59.7)	NR	504 (35)	816 (56.7)	173 (12)	96 (6.6)	259 (18)	NR	NR
Cavalcanti	269	50.3 (14.6)	157 (58.3)	NR	51 (18.9)	104 (38.7)	11 (12)	4 (1.5)	21 (7.8)	2 (0.7)	NR

**Abbreviations:** N, n: number of patients, BMI: basic metabolic index, HTN: hypertension, CAD: coronary artery disease, CHF: congestive heart failure, COPD: chronic obstructive pulmonary disease, CKD: chronic kidney disease, ESRD: end stage renal disease, NR: not reported, QTc: corrected QT. * out of 55 patients with available data.

**Table 3:** Incidence of arrhythmias and torsades de point reported by the included studies.

Study	Torsades De Point n (%)	Ventricular Arrhythmia n (%)
Van den Broek	0 (0)	0 (0)
Sinkeler	NR	1 (0.2)
Mercuro	1 (11)	NR
Saleh	0 (0)	7 (3.7) (non-sustained) 1 (0.5) (sustained)
Bessiere	0 (0)	0 (0)
Enzmann	NR	14 (18.7)
Tuncer	0 (0)	0 (0)
Moschini	1 (11)	2 (1.8)
Borba	NR	NR
Chorin	1 (0.4)	NR
Maraj	1 (11)	2 (2)
Cipriani	NR	NR
Ramireddy	0 (0)	NR
Kelly	NR	NR
Samuel	0 (0)	6 (16.7)
Lagier	0 (0)	NR
Rosenberg	NR	NR
Cavalcanti	NR	NR

**Abbreviations:** NR: not reported.
